# Monocarboxylate transporter-1 (MCT1) protein expression in head and neck cancer affects clinical outcome

**DOI:** 10.1038/s41598-021-84019-w

**Published:** 2021-02-25

**Authors:** Martin Leu, J. Kitz, Y. Pilavakis, S. Hakroush, H. A. Wolff, M. Canis, S. Rieken, M. A. Schirmer

**Affiliations:** 1 Clinic of Radiotherapy and Radiation Oncology, University Medical Center Göttingen, Robert-Koch-Strasse 40, 37075 Göttingen, Germany; 2 Institute of Pathology, University Medical Center Göttingen, Robert-Koch-Strasse 40, 37075 Göttingen, Germany; 3 Clinic of Otorhinolaryngology, University Medical Center Göttingen, Robert-Koch-Strasse 40, 37075 Göttingen, Germany; 4 Department of Radiology, Nuclear Medicine and Radiotherapy, Radiology Munich, Maximiliansplatz 2, 80333 Munich, Germany; 5 Department of Radiation Oncology, Medical Center, University of Regensburg, Franz-Josef-Strauss-Allee 11, 93053 Regensburg, Germany; 6 Department of Otorhinolaryngology, Head and Neck Surgery, Ludwig-Maximilians-University Munich, Marchioninistrasse 15, 81377 Munich, Germany

**Keywords:** Head and neck cancer, Cancer, Biomarkers, Prognostic markers, Oncology, Cancer, Head and neck cancer, Tumour biomarkers

## Abstract

Treatment of locally advanced, unresectable head and neck squamous cell carcinoma (HNSCC) often yields only modest results with radiochemotherapy (RCT) as standard of care. Prognostic features related to outcome upon RCT might be highly valuable to improve treatment. Monocarboxylate transporters-1 and -4 (MCT1/MCT4) were evaluated as potential biomarkers. A cohort of HNSCC patients without signs for distant metastases was assessed eliciting 82 individuals eligible whereof 90% were diagnosed with locally advanced stage IV. Tumor specimens were stained for MCT1 and MCT4 in the cell membrane by immunohistochemistry. Obtained data were evaluated with respect to overall (OS) and progression-free survival (PFS). Protein expression of MCT1 and MCT4 in cell membrane was detected in 16% and 85% of the tumors, respectively. Expression of both transporters was not statistically different according to the human papilloma virus (HPV) status. Positive staining for MCT1 (n = 13, negative in n = 69) strongly worsened PFS with a hazard ratio (HR) of 3.1 (95%-confidence interval 1.6–5.7, p < 0.001). OS was likewise affected with a HR of 3.8 (2.0–7.3, p < 0.001). Multivariable Cox regression confirmed these findings. We propose MCT1 as a promising biomarker in HNSCC treated by primary RCT.

## Introduction

Head and neck squamous cell carcinoma (HNSCC) comprising manifestations in the oral cavity, oropharynx, hypopharynx, and larynx is placed seventh among cancer incidence worldwide^1^. Based on contemporary data, this entity affects 64,000 and 140,000 patients per annual incidence and 14,500 and 63,500 per mortality in the US and Europe, respectively^1–3^. Metastasis to locoregional lymph nodes is regarded the most accurate prognostic factor of clinical outcome^4^. In addition, positive human papilloma virus (HPV) status has been established as a major prognostic factor with a relative death risk reduced by more than 50%^5,6^ and is thus increasingly considered for clinical management. Positivity for p16 protein staining is often used as a proxy for HPV status^7^. Beyond the HPV status, which affects roughly about 40% of HNSCC cases, there is a high demand for further biomarkers. This particular refers to patients with HPV negative tumors typically associated with smoking and/or alcohol consumption.

Due to high proliferation rates malignant tumors are typically confronted with lack of nutrients and contain admixtures of normoxic and hypoxic cells. Malignant cells have adopted mechanisms to overcome these obstacles. One important issue concerns lactate exchange between tumor cells. The identification of this mechanism represents a milestone in the field^8–10^: hypoxic cancer cells provide lactate used as energy source for mitochondrial respiration by their counterparts supplied with sufficient oxygen termed as “metabolic symbiosis”. Tumor-associated cells might also cooperate in this well-orchestrated process^11^. Lactate utilization seems to be a particular feature of aggressive tumor behavior^12^. Elevated tumor lactate concentrations were reported to be linked with increased risks for disease recurrence and metastasis also in HNSCC^13,14^.

By virtue of its negative charge lactate requires specific proteins for transport through the cell membrane^15^. Monocarboxylate transporters (MCTs), also termed as solute carrier family 16 A (SLC16A), comprise 14 homologues in mammals^16–19^. They facilitate passive transport of unbranched alipathic monocarboxylate acids—as symport with protons or as exchange with another monocarboxylate anion—over cell membranes^20,21^. MCT1 and MCT4 represent the most extensively studied monocarboxylate transporters in human cancer. They have been identified as the major players in the context of lactate exchange in tumor tissue. In the cytoplasm of tumor cells, lactate concentrations can reach concentrations up to 40 mM^22–24^.

MCT1 (alias SLC16A1) acts bi-directionally, dependent on substrate gradient, features a high affinity for lactate and is expressed in almost all cell types^25–28^. This transporter is regarded as the major route for lactate uptake of malignant cells which use lactate as metabolic fuel^10,12^.

MCT4 (alias SLC16A3) has a lower affinity for lactate than MCT1 and predominantly effectuates lactate efflux out of hypoxic cells^29,30^. Unlike MCT1, MCT4 is up-regulated by hypoxia^31^.

Expression of MCT1 and MCT4 in cancer was frequently associated with poor clinical outcome in a variety of cancers including gynecological, esophageal, gastro-intestinal, and urologic entities^23,24,32–37^. In HNSCC, a pioneer study suggested a negative impact of MCT1 and MCT4 expression in relation to clinical outcome^38^. Further evidence for clinical significance was provided for MCT1 along with mechanistical investigations^39^. Despite the hypothesis from the two denoted studies suggesting a prognostic relevance of these transporters in HNSCC, its clinical significance remains to be proven. They have been proposed as functional biomarkers of the above-described “metabolic symbiosis” in HNSCC^40^. We set up to study protein expression of these two transporters in biopsy specimens from HNSCC tumors of patients prior to be subjected to primary radio(chemo)therapy with curative treatment intention. The hypothesis tested was that MCT1 and/or MCT4 may affect clinical outcome in terms of PFS and OS.

## Results

### Baseline patient and clinical parameters

**Figure 1:**
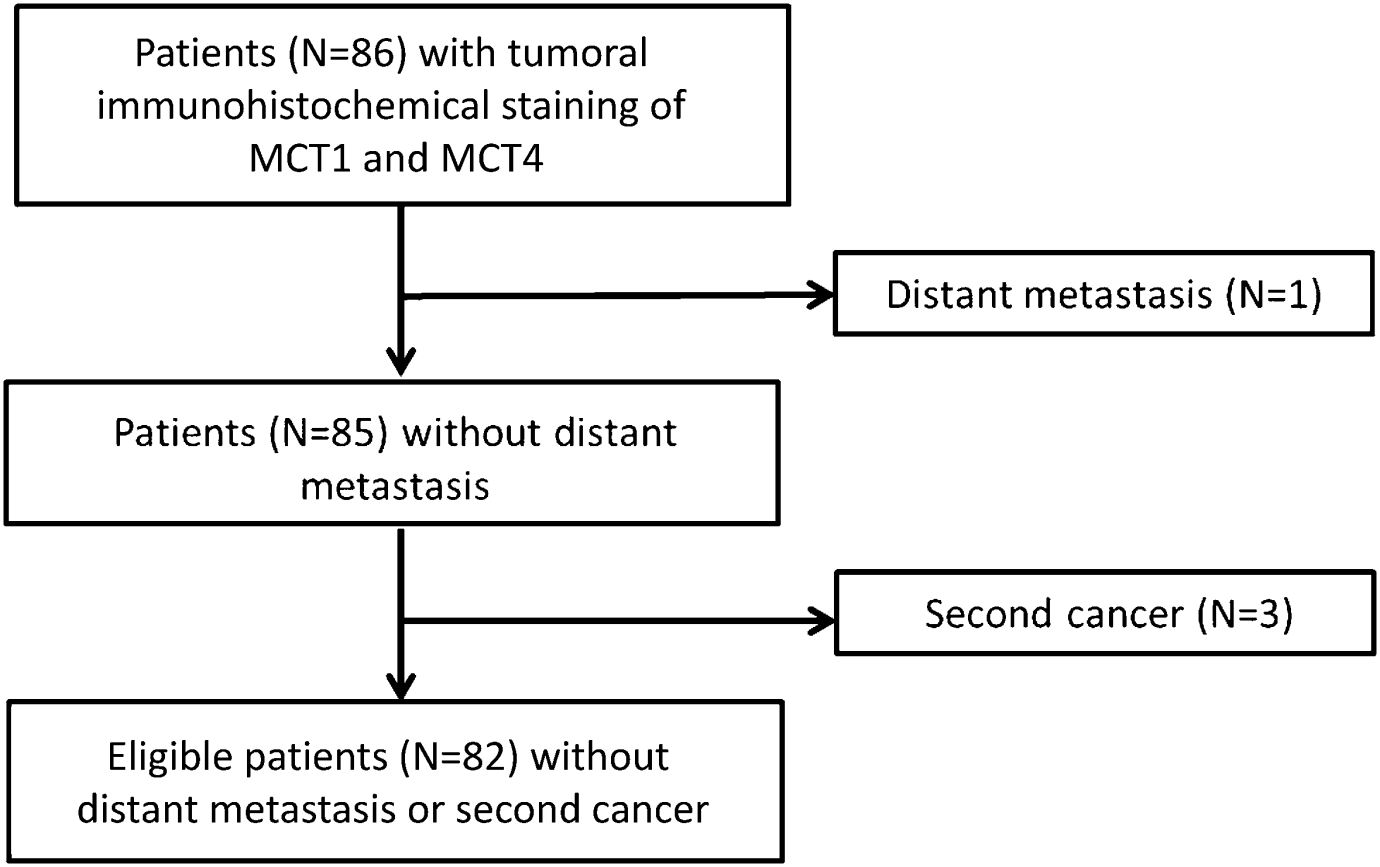
Flowchart for patients eligible for study.

For eligible patients (see flowchart Fig. 1 in “Methods”), baseline parameters characterizing patient, tumor and treatment features are summarized in Table 1. Most of the tumors presented in inoperably advanced stages in line with indication for primary radio(chemo)therapy. HPV status was assessed as both positivity for DNA of the high-risk subtype HPV-16 and p16 protein staining. The high proportion of samples immunohistochemically positive for p16 suggests substantial involvements of subtypes other than HPV-16.

**Table 1:** Baseline patient and disease characteristics.

Patient and disease characteristics, N (%)	All eligible patients (n = 82)
Age, median (min, max)	56.4 (20.7, 88.8)
**Sex**	
Female	15 (18.3)
Male	67 (81.7)
**Behavioral factors**	
Smoking w/o regular alcohol	16 (25.4)
Alcohol abuse w/o smoking	2 (2.4)
Smoking and alcohol abuse	52 (63.4)
Neither smoking nor regular alcohol	6 (7.3)
Undetermined	6 (7.3)
**Tumor site**	
Oral cavity	24 (29.3)
Oropharynx	37 (45.1)
Hypopharynx	14 (17.1)
Larynx	7 (8.5)
**History of former HNSCC**	
No	71 (86.6)
Yes	11 (13.4)
**T category clinical**	
T1	5 (6.1)
T2	5 (6.1)
T3	11 (13.4)
T4	61 (74.4)
**Nodal status clinical**	
N0	10 (12.2)
N1	9 (11.0)
N2	57 (69.5)
N3	6 (7.3)
**Disease stage**	
SI	0 (0.0)
SII	3 (3.7)
SIII	5 (6.1)
SIV	74 (90.2)
**Tumor grade**	
G1	3 (3.6)
G2	64 (78.1)
G3	15 (18.3)
**HPV assessment**	
HPV-16 DNA positivity	16 (19.5)
p16 protein positivity	40 (48.8)
**Radiation dosage (% achieved of planned)**	
100%	73 (89.0)
> 70 to < 100%	9 (11.0)
**Concomitant chemotherapy**	
No	18 (22.0)
Yes	64 (78.0)

### Expression distribution and inter-rater reliability of MCT1 and MCT4

Examples for immunohistochemical staining are provided in Supplemental Fig. 1 (MCT1) and 2 (MCT4). Frequencies of expression distribution patterns in the 82 samples are depicted as histograms in Supplemental Fig. 3. Only 13 (16%) samples showed positive staining of MCT1 expression in tumor cell membranes confirmed by both pathologists. Thus, the “Cohens Kappa” was 1.0 for MCT1 dichotomized in positive *versus* negative, i.e. demonstrating maximum concordance. This dichotomization was suggested by an online available tool (“Cutoff Finder”, see “Methods” below). When the raw MCT1 staining data as continuous variable was considered, Kendall’s tau was 0.996 indicating also very high conformity.

In contrast to MCT1, MCT4 expression in tumor cell membrane was more common. According to both pathologists MCT4 was rated positive in 70 (85%) samples and was absent in 12. Considering the raw continuous MCT4 membrane staining data the Kendall’s tau amounted to 0.993. Unlike MCT1, dichotomization defined by “Cutoff Finder” elicited specific thresholds for MCT4 (“high” versus “low”) in regard to PFS and OS (see "Methods"). The “Cohens Kappa” was both 1.0 when the dichotomization cut-offs for PFS and OS were used. Thus, there were no inter-rater discrepancies affecting the statistical analysis.

MCT1 and MCT4 staining did not significantly differ when stratified by HPV status (assessed as positivity for high risk subtype HPV-16 DNA and p16 protein, all p > 0.1, Mann–Whitney U test).

### MCT1 and MCT4 in relation to PFS and OS

**Figure 2:**
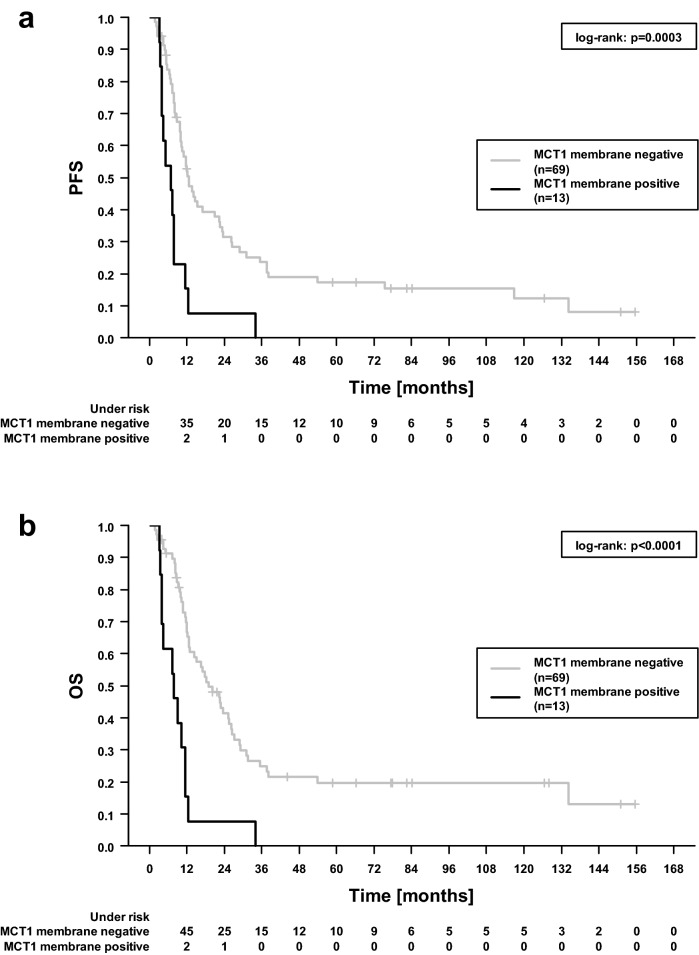
Progression-free (**a**) and overall survival (**b**) dependent on MCT1 membrane tumor cell status. The denoted p values refer to log-rank test.

**Figure 3:**
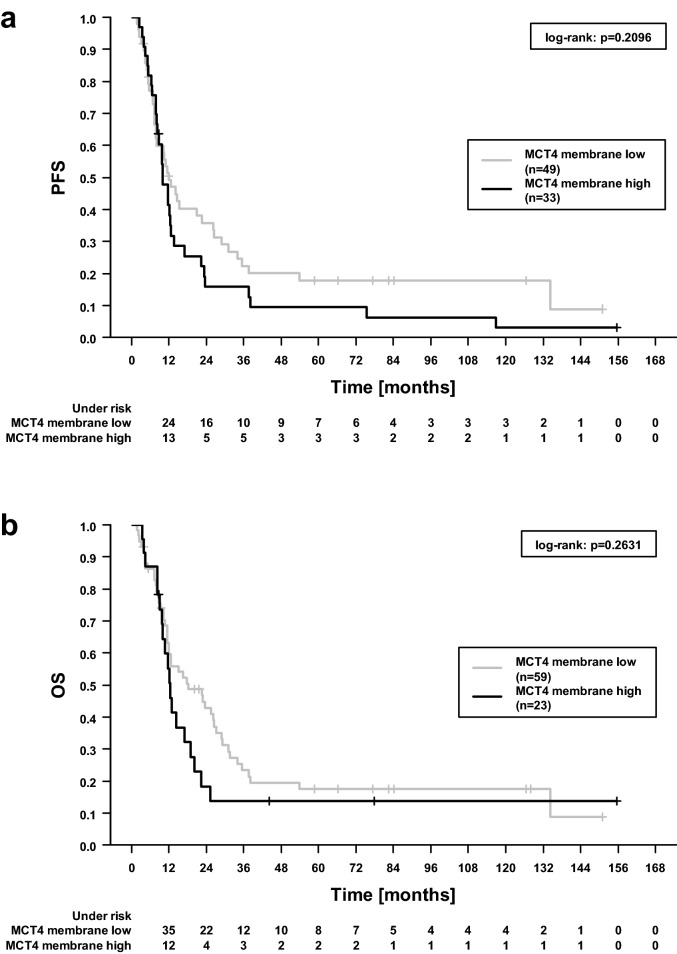
Progression-free (PFS, **a**) and overall survival (OS, **b**) dependent on MCT4 membrane tumor cell status; “high” denotes an immunohistochemical score ≥ 165 and ≥ 215 regarding PFS and OS, respectively. The denoted p values refer to log-rank test.

Membrane-associated MCT1 exhibited a highly significant impact on PFS (p = 0.0003, log-rank test) and, even more pronounced, on OS (p = 1 × 10^–5^), see Fig. 2. Median PFS was 12.6 and 6.8 months when MCT1 staining was negative and positive with a five-year PFS rate of 18.4% and 0%, respectively. Likewise, median OS was 20.1 and 8.0 months and 5-year OS 21.4% and 0%. In this univariable analysis, no statistically significant associations of MCT4 with either PFS (p = 0.2, Fig. 3a) or OS (p = 0.2, Fig. 3b) were observed.

Univariable Cox regression confirmed a strong impact of MCT1 expression on outcome (Table 2). Patients harboring tumors with positive MCT1 staining encountered a more than threefold hazard ratio for worse PFS or OS. A beneficial effect was noticed for tumors positive for p16 protein used as a proxy for HPV infection.

**Table 2:** Basic patient and clinical features in relation to progression-free (PFS) and overall (OS) survival assessed by univariable Cox regression analysis.

Variable	PFS		OS	
	Hazard ratio (95% CI)	p value	Hazard ratio (95% CI)	p value
Age (per year)	1.00 (0.98–1.02)	0.758	1.00 (0.98–1.02)	0.706
**Sex**				
Female (15) vs male (67)	0.94 (0.51–1.76)	0.851	1.05 (0.55–2.00)	0.895
**T stage**				
T4 (61) vs T1–T3 (21)	0.90 (0.53–1.52)	0.686	0.88 (0.50–1.53)	0.642
**N stage**				
N+ (72) vs N0 (10)	0.96 (0.48–1.95)	0.917	1.18 (0.54–2.59)	0.687
N ≥ 2 (63) vs N < 2 (19)	0.85 (0.49–1.45)	0.544	0.82 (0.46–1.45)	0.492
**Grading**				
G3 (15) vs G1–G2 (67)	1.14 (0.62–2.08)	0.678	1.34 (0.73–2.47)	0.348
**HPV status**^**a**^				
p16^+^ (40) vs p16^−^ (42)	0.56 (0.35–0.91)	**0.018**	0.50 (0.31–0.83)	**0.007**
DNA^+^ (16) vs DNA^−^ (66)	0.77 (0.41–1.45)	0.421	0.64 (0.31–1.29)	0.212
**Radiotherapy technique**				
IMRT + VMAT (20) vs conventional (62)	0.94 (0.53–1.64)	0.817	0.76 (0.42–1.38)	0.368
**Radiotherapy completed**				
Yes (73) vs no (9)	0.84 (0.39–1.85)	0.670	0.79 (0.34–1.83)	0.575
**Chemotherapy administered**				
Yes (64) vs no (18)	0.64 (0.37–1.11)	0.114	0.62 (0.35–1.10)	0.101
**History of former HNSCC**				
Yes (11) vs no (71)	1.02 (0.52–2.00)	0.949	0.95 (0.47–1.93)	0.892
**MCT1**				
Positive (13) vs negative (69)^b^	3.07 (1.64–5.73)	**4 × 10**^**–4**^	3.84 (2.03–7.26)	**4 × 10**^**–5**^
**MCT4**				
High (33) vs low (49)^b^	1.35 (0.84–2.17)	0.210		
High (23) vs low (59)^b^			1.39 (0.81–2.39)	0.230

P values < 0.05 are indicated in bold.

^**a**^HPV status was determined both by p16 protein and specifically for HPV-16 subtype by DNA sequencing. Note that other HPV viruses were not covered by this sequencing.

^b^Dichotomic discriminator was determined by “Cutoff Finder”, see “Methods” section. For MCT4, different cutoffs were determined for PFS (score 165 as defined in “Methods”) and OS (215).

In multivariable Cox regression analyses (Table 3), the strong link of positive MCT1 staining with worse PFS (hazard ratio 3.36, 95% confidence interval 1.60–7.10, p = 0.001) and OS (4.38, 1.97–9.74, p = 3 × 10^–4^) was confirmed. In these multivariable models, more intense staining of MCT4 showed a borderline association with shorter PFS (1.66, 0.97–2.83, p = 0.063) and OS (1.83, 0.96–3.46, p = 0.066).

**Table 3:** Basic patient and clinical features in relation to progression-free (PFS) and overall (OS) survival assessed by multivariable analysis including all the designated variables.

Variable	PFS		OS	
	Hazard ratio (95% CI)	p value	Hazard ratio (95% CI)	p value
Age (per year)	1.00 (0.97–1.02)	0.579	1.00 (0.97–1.02)	0.820
**Sex**				
Female (15) vs male (67)	1.02 (0.53–1.96)	0.949	1.00 (0.50–2.01)	1.000
**T stage**				
T4 (61) vs T1-T3 (21)	0.76 (0.42–1.38)	0.367	0.60 (0.31–1.18)	0.141
**N stage**				
N ≥ 2 (63) vs. N < 2 (19)	0.83 (0.47–1.48)	0.532	0.77 (0.42–1.42)	0.397
**Grading**				
G3 (15) vs G1–G2 (67)	1.46 (0.72–2.98)	0.294	1.76 (0.84–3.68)	0.134
**HPV status**				
p16^+^(40) vs p16^-^(42)	0.62 (0.35–1.09)	0.099	0.55 (0.31–0.99)	**0.045**
**Radiotherapy technique**				
IMRT + VMAT (20) vs conventional (62)	1.21 (0.63–2.33)	0.571	0.96 (0.47–1.94)	0.907
**Radiotherapy completed**				
Yes (73) vs no (9)	0.49 (0.20–1.22)	0.125	0.37 (0.14–0.98)	**0.045**
**Chemotherapy administered**				
Yes (64) vs no (18)	0.66 (0.33–1.31)	0.233	0.64 (0.31–1.32)	0.228
**History of former HNSCC**				
Yes (11) vs no (71)	0.80 (0.32–1.96)	0.623	0.61 (0.24–1.54)	0.291
**MCT1**				
Positive (13) vs negative (69)^a^	3.07 (1.44–6.58)	**0.004**	3.79 (1.68–8.58)	**0.001**
**MCT4**				
High (33) vs low (49)^a^	1.48 (0.85–2.58)	0.172		
High (23) vs low (59)^a^			1.70 (0.89–3.26)	0.108

P values < 0.05 are indicated in bold.

^a^Cutoff determined by “Cutoff Finder”. For MCT4, different cutoffs were determined for PFS (score 165 as defined in “Methods”) and OS (215).

### MCT1 and MCT4 in dependence on tumor size

The subgroup with stage T4 tumors was most prominent in this patient cohort treated primarily with RCT for advanced disease stage. T4 versus T1-3 tumors were accompanied with higher MCT4 membrane expression (p = 0.03 according to Mann–Whitney U test) whereas no such relation was seen for MCT1 (p = 0.3). Tumor grading did not significantly affect MCT1 or MCT4 expression status (p ≥ 0.1). The most advanced disease stages (i.e. IV) did not present with statistically elevated levels of MCT1 or MCT4 (p ≥ 0.1).

### MCT1 and MCT4 in dependence on tumor sites

Distribution of MCT1 and MCT4 did not differ by the anatomical tumor sites (oral cavity, oropharynx, hypopharynx, larynx; p = 0.4 for both transporters according to Kruskal–Wallis test). When considered in relation to clinical outcome, MCT1 expression was linked to worse PFS and OS in oropharyngeal cancer, which represents the largest subgroup in our cohort, at p = 0.008 and 0.001, respectively. The second most frequent localization was oral cavity, for which MCT1 was related at p = 0.07 and 0.02 with PFS and OS. Regarding hypopharynx and larynx, only one PFS and OS event each occurred not rendering statistical analyses reasonable.

### Prognostic effects of MCT1 in relation to HPV status

Positivity of p16 was used as surrogate for HPV infection as direct viral DNA proof was available only for the subgroup of high risk HPV-16. In our sample, we did not observe statistically significant distinctions of MCT1/4 expression in dependence on the HPV/p16 status though MCT1 positivity was slightly over-represented in p16-negative (9 out of 33 samples) *vs* p16-positive (4/36) tumors (p = 0.13 for the latter, Fisher’s exact test). Interestingly, it revealed that the effect of MCT1 on outcome was much more pronounced in the subgroup of p16 positive samples. A poor prognosis, in terms of PFS and OS, was noticed for the very few tumors with p16+ and detectable MCT1 defining a subgroup of patients with worse outcome despite p16+ (see Supplemental Fig. 4). When stratified by MCT1 status, p16 positivity only turned out beneficial in MCT1 negative tumors, though one should consider the low numbers of MCT1 positive tumors (Supplemental Fig. 5). This prompted us to define a subgroup of patients with a particular favorable outcome when the tumors were positive for p16 and simultaneously negative for MCT1 (Supplemental Fig. 6). In other words, the beneficial effect clinically established for p16 is not valid when MCT1 is detectable in parallel.

## Discussion

This study revealed MCT1 as a promising biomarker for disease progression and overall survival in a human cohort with HNSCC treated with definitive RCT without surgical tumor resection. The prognosis was worsened by hazard ratios of more than three-fold in regard to both PFS and OS if tumor cell membrane staining was positive for MCT1. In contrast, the hypothesis that the extent of MCT4 expression in tumor might also affect patient outcome could not be proven. Considering other patient-, tumor- and treatment-related features only MCT1 and HPV status, turned out associated with prognosis. Interestingly, the established clinical beneficial feature of HPV association assessed as p16 positivity as a surrogate seems to be valid only when MCT1 is not expressed in parallel. This finding might be of high clinical relevance when treatment stratification might become based on the HPV status. As a result of this study, MCT1 testing should be indispensably included in future conceptualizations. The prognostic effect of MCT1 is probably valid for all primary HNSCC sites, although the sample numbers in our cohort were too small to outline this for the anatomical subgroups.

The first pioneering study in this field was performed by Simões-Sousa et al.^38^. Whereas they did not find an effect of MCT transporter expression on disease-free survival they reported an impact on OS with an association at p = 0.044 only for a specific combination of MCT1 (positive), MCT2 (negative), and MCT4 (positive). However, due to multiplicity testing considerations this association might be regarded rather as hypothesis generating than proving. The latter might similarly also apply to a recent study in which immunohistochemical MCT1 expression was attributed a negative impact on disease-free survival in oropharyngeal HNSCC^39^. That study refers to a combined cohort of patients treated primarily either by surgery or RCT. The effect of MCT1 on disease-free survival was mainly observed in the subgroup of 48 patients treated with primary RCT, comparable to our findings, rather than in those having undergone primary resection. We could confirm the unfavorable effect of MCT1 tumor protein expression on disease progression and suggest that this relation extends on OS as well. In contrast, such relations were not reported for other members of the monocarboxylic acid transporter family analyzed in the cited study^39^ consistent with absence of any impact of MCT4 on clinical outcome in our investigation.

MCT1 expression appears to be a negative feature in other cancer entities as well^23,24^. Upregulation of MCT1 was found in breast cancer subtypes with poor prognosis, in particular the basal-like phenotype^34^. In ovary cancer, MCT1 and MCT4 were not correlated with histology but with tumor progression^32^. A direct link between MCT1 tumor cell membrane expression and vascular invasion was noticed in colon cancer^35^. Unlike MCT4, increased expression of MCT1 was connected with advanced stages of stomach cancer^36^. Likewise, MCT1 might be a biomarker for reduced PFS and OS in esophageal squamous cell carcinoma as well^33^, a tumor entity closely related with HNSCC in terms of etiology and histopathologic features. Shorter OS along with enhanced metastasis was observed also for high MCT1 expression in urinary bladder cancer^37^. Contrarily, in pancreatic ductal adenocarcinoma MCT1 expression might come along with better prognosis and reduced nodal metastasis^41^.

Functional analyses conducted in the above-mentioned reference^39^ also revealed that inhibition of MCT1 resulted in suppressed tumor cell invasion, decreased colony formation, and enhanced radiosensitivity in a cell culture model. These findings convey plausible explanations for the particular relevance of MCT1 expression in primary RCT. This fits well to a milestone study, in which inhibition of MCT1 led to a shift of lactate-fueled respiration toward aerobic glycolysis in oxygenated tumor cells along with enhanced and reduced consumption of glucose and oxygen, respectively. Consecutively, hypoxic cells died from glucose starvation and the surviving tumor areas became more sensitive toward irradiation due to better oxygenation following less oxygen consumption^10^. In a subsequent study using a more specific inhibitor of MCT1 in combination with irradiation, this mechanism was confirmed. As a result of reduced glucose bioavailability levels of ATP were lower and those of reactive oxygen species higher in hypoxic tumor regions^42^. These observations led to the hypothesis of additive effects of MCT1 inhibition and radiotherapy on tumor cell killing. However, it should be noted that the molecular interplays between MCT1 and radiotherapy are yet based on only sparse date requiring further investigation. What already seems clear is that cancer cells may profit in either way from adaptive up-regulation of MCT1 expression. Hypoxic cells can get rid of deleteriously high lactate concentrations which in turn can be used as an energy supply by cells capable to oxidative respiration. These mechanisms might explain the negative impact of MCT1 expression on patient outcome observed in our study. At present, it cannot be determined whether MCT1 also may act as a predictive biomarker for the effects of RCT in addition to its established feature of worsening disease prognosis.

Blocking lactate acid export of hypoxic cells was also suggested as an effective anticancer strategy^43,44^. According to our observation, the inverse relation of MCT1 expression with clinical outcome more probably relies on its relevance for lactate uptake by oxygenated cells. In the cited study of Le Floch et al. the anti-tumor effect of MCT1 silencing could be reversed by restoration of MCT4 expression. Unlike MCT1, MCT4 is hypoxia-inducible and should thus prevail in hypoxic cells. Larger tumors are more likely to contain hypoxic areas. Concordantly, we found higher MCT4 expression in tumors staged as T4 in comparison to T1-T3. However, we could not demonstrate a clear effect of MCT4 on outcome though its protein expression was much more abundant than that of MCT1 in our sample. This is also different to a report having found a worse prognosis of MCT4 expression in HNSCC restricted to the oral cavity^45^.

HPV status, assessed as positive p16 protein staining, turned out as a prognostic feature at a nominal statistical threshold of p < 0.05 for PFS and OS in univariable and for OS in multivariable Cox regression analyses. The beneficial impact of HPV positivity on prognosis has become an established feature in clinical routine^5,6^ though consequences for treatment remain to be clarified pending prospective clinical trials. Regardless the above-mentioned interaction in terms of clinical outcome, we did not observe a statistically significant relation between HPV/p16 and MCT1/MCT4 expression. The study of Fleming et al.^39^ has reported distinct metabolic profiles with significantly higher MCT1 in HPV-negative tumors.

Limitations of our study are its sample size together with the retrospective nature of the investigation making it sensitive to uncontrolled factors potentially biasing the findings. However, as far as possible, we analyzed for tumor-, patient-, and treatment-related parameters. Besides tumoral MCT1 protein expression only HPV status, assessed as p16 positivity, exhibited an impact on clinical outcome (Table 2).

A strength of our study is that it shows an impact of MCT1 expression on clinical outcome in HNSCC, for the first time, in a statistically highly significant fashion even when considering multiple testing. Another important point of our study is that it refers to a clearly defined treatment. Though not all patients received concomitant chemotherapy and some patients did not achieve the initially planned cumulative radiation dosage this study is the first focused on definitive RCT in HNSCC, for which a borderline association was reported previously^39^.

Pretherapeutic histopathologic assessment of MCT1 status in tumor specimens is an achievable task comparable to HPV staining. According to our data the fraction of tumors positive for MCT1 is relatively small limiting possible additional efforts (e.g. intensified after-care, complementary diagnostics) subjected to this subgroup. Based on current data and strongly supported by this study a negative impact of high MCT1 seems clinically relevant for primary RCT. This is possibly not the case for patients subjected to primary resection as previously suggested^39^. If the latter is independently confirmed MCT1 expression status could become a valuable parameter for treatment tailoring: Surgery, if technically feasible, may thus be preferred for tumors with high MCT1 content.

Aside this stringent clinical relevance, the here presented findings should stimulate further mechanistic research to better decipher cross-talks between MCT1 expression and effects of radiotherapy and/or chemotherapy. Beyond that defined clinical and therapeutic entity, further molecular exploration of MCT1 regulation and targeting might put forth new strategies to enhance cure rates of locally advanced unresectable HNSCC, a cancer entity whose treatment often results in unsatisfactory results.

## Methods

### Eligible patients

Searching clinical records and pathology archives at a single institution (Göttingen University Medical Center) from 08/1995 to 08/2012 identified 86 patients consecutively treated for HNSCC with primary HNSCC, for which sufficient formalin-fixed paraffin-embedded (FFPE), pretreatment tumor tissue for immunohistochemical staining was available. Patients with metastatic disease or formerly diagnosed for another cancer were excluded (see flowchart Fig. 1) resulting in 82 eligible patients. They were diagnosed with untreated, pathologically confirmed HNSCC of the oral cavity, oropharynx, hypopharynx or larynx classified as UICC (Union for International Cancer Control) stages II, III or IV without distant metastasis at diagnosis. One of these 82 patients presented with a cT4 cN2 carcinoma of the hypopharynx who underwent surgical tumor debulking resulting in a R2 resection and later received definitive RCT. Patient characteristics are summarized in Table 1. For treatment modalities, see “Methods” section below.

### Treatment modalities

Patients of this study are part of a cohort, for which treatment modalities were previously described^46^. Briefly, 62 patients, from 08/1995 to 08/2012, received normofractionated definite radiotherapy (RT) as parallel-opposed lateral portals (2.0 Gy/day, 5 times/week). For 17 patients, from 07/2008 to 06/2011, an integrated intensity-modulated radiotherapy (IMRT) with single fractions of 2.2 Gy to the primary tumor and involved lymph nodes up to 66 Gy and single fractions of 1.8 Gy to the drainage sites on both sides of the neck up to 54 Gy was applied daily (5 times/week). For three patients, from 11/2009 to 10/2011, an integrated volumetric modulated arc radiotherapy (VMAT) with single fractions of 2.2 Gy to the primary tumor and involved lymph nodes up to 66 Gy and single fractions of 1.8 Gy to the drainage sites on both sides of the neck up to 54 Gy was delivered daily (5 times/week). The majority of patients (n = 64) received concomitant radiochemotherapy (RCT). Chemotherapy consisted either of 5-fluorouracil plus mitomycin C (n = 34), cisplatin only (27) or of cetuximab (3).

Out of the entire cohort comprising 82 patients, 73 received the full radiation dose as initially prescribed. The remaining nine, of whom seven were treated with concomitant chemotherapy, did not reach the full RT dose for the following reasons: One patient died from tumor progress when having received 54 Gy (planned 70 Gy). Two patients refused further treatment at a dose of 54 and 52 Gy (70 Gy). In six patients, treatment was stopped by a radiation oncologist’s decision at doses ≥ 62 Gy (70 Gy): four due to side effects from radiotherapy, one with chemotherapy-related thrombocytopenia IV° accompanied by clinically relevant bleeding, and one who presented considerable deterioration of general health state. The portion of patients without concomitant chemotherapy as a radiosensitizer was very similar between those who reached full RT dose (15/73, 21%) in comparison to those without RT completion (2/9, 22%). In addition, as MCT1 and MCT4 expression represents a tumor and not treatment feature all 82 patients were considered for further statistical analysis.

### Patient follow-up

Median follow-up in this cohort with advanced disease stages along with poor prognosis was 13.7 months (range 1.8–156). Following RCT, patients underwent quarterly clinical ear-nose-throat examination, chest radiography, or contrast-enhanced CT of the head and neck, if necessary. Complete remission was defined as the complete regression of all tumor manifestations. Biopsy specimens were taken from suspect findings to receive histologic confirmation of tumor (re)growth.

### Immunohistochemistry

Immunohistochemical staining of MCT1 and MCT4 was performed on FFPE tissue samples from pretreatment tumor biopsies. A standardized immunohistochemical staining technique was performed using polyclonal rabbit-anti-MCT1 (H-70:sc-50324; Santa Cruz Biotechnology, Inc., Santa Cruz, USA) and polyclonal rabbit-anti-MCT4 (H-90:sc-50329; Santa Cruz Biotechnology, Inc., Santa Cruz, USA) antibodies on a Ventana BenchMark XT immunostainer (Ventana, Tucson, AZ). Staining and evaluation procedures were carried out as reported previously^46^. Heat epitope retrieval using the immunostainer was performed for 60 min at 100 °C. The antibodies were incubated at 37 °C for 32 min. The staining reaction was visualized by means of horseradish peroxidase with the ultraView Universal DAB Detection Kit (Ventana Medical Systems) and hematoxylin solution (Gill 3, Sigma Aldrich, Munich, Germany) for counterstaining. Negative control slides in the absence of primary antibodies were included for each staining. Immunoreactivity was evaluated taking into account the fraction of positive cells and the intensity of staining. The staining intensity was scored as: 1+ (weak), 2+ (moderate) and 3+ (intense). The individual weighted labeling score for MCT1 and MCT4 results from the addition of the products of the percentage of positive tumor cells multiplied by their staining intensity, thus scores from 0 (no positive tumor cell) to 300 (100% intensely stained tumor cells) could be obtained.

Two registered senior specialists in pathology performed the immunohistochemical evaluation. They were both blinded for the clinical patient courses. To assess the inter-rater reliability, we applied “Cohens Kappa” for dichotomized data. Evaluation of inter-rater variability on continuous staining data was ascertained by Kendall’s tau. For both approaches, values may reach from − 1.0 (complete discordance) to 1.0 (complete concordance).

### Statistical analysis

Patient baseline, tumor, treatment and immunohistochemical data were analyzed with respect to OS and PFS by means of univariate Cox proportional hazards regressions. Survival times were calculated from the day of pathologically determined malignancy diagnosis until the end of follow-up. To evaluate the effects of MCT1 and MCT4 expression on survival parameters immunohistochemistry scoring data were dichotomized using “Cutoff Finder” (http://molpath.charite.de/cutoff/)^47^. Furthermore, multivariable Cox regression was performed to test whether the association of the investigated immunohistochemical marker expression and survival was independent from other factors possibly affecting treatment outcome. Visual illustration of MCT1 and MCT4 in regard to OS and PFS was performed by Kaplan–Meier plots along with log-rank statistics. Each statistical test was performed with a significance level of α = 5%. Data were analyzed using the software SPSS Statistics (version 26, IBM) and R 4.0.2 with “KMWin” plugin for Kaplan–Meier plots^48^.

### Ethics approval

Our investigation with use of histopathological specimen and clinicopathological data was approved by the local ethic committee of the University of Goettingen Medical Center (approval code “22/8/20”) and performed in accordance with the WMA Declaration of Helsinki and the Department of Health and Human Services Belmont Report. All clinical samples were obtained upon written informed consent during routine surgery or biopsy.

## Supplementary Information

Supplementary Figures.

## Data Availability

The datasets, on which the current manuscript is based, are available from the corresponding author on reasonable request.

## Abbreviations

HNSCC: Head and neck squamous cell carcinoma
OS: Overall survival
PFS: Progression-free survival
RCT: Radiochemotherapy
HR: Hazard ratio
CI: Confidence interval
MCT: Monocarboxylate transporter
HPV: Human papilloma virus

## References

[1] Ferlay J (2013). Cancer incidence and mortality patterns in Europe: Estimates for 40 countries in 2012. Eur. J. Cancer.

[2] Gatta G (2015). Prognoses and improvement for head and neck cancers diagnosed in Europe in early 2000s: The EUROCARE-5 population-based study. Eur. J. Cancer.

[3] Siegel RL, Miller KD, Jemal A (2020). Cancer statistics, 2020. CA Cancer J. Clin..

[4] Marur S, Forastiere AA (2016). Head and neck squamous cell carcinoma: Update on epidemiology, diagnosis, and treatment. Mayo Clin. Proc..

[5] Ang KK (2010). Human papillomavirus and survival of patients with oropharyngeal cancer. N. Engl. J. Med..

[6] Fakhry C (2008). Improved survival of patients with human papillomavirus-positive head and neck squamous cell carcinoma in a prospective clinical trial. J. Natl. Cancer Inst..

[7] Gillison ML (2012). Tobacco smoking and increased risk of death and progression for patients with p16-positive and p16-negative oropharyngeal cancer. J. Clin. Oncol..

[8] Bonuccelli G (2010). Ketones and lactate "fuel" tumor growth and metastasis: Evidence that epithelial cancer cells use oxidative mitochondrial metabolism. Cell Cycle.

[9] Kennedy KM (2013). Catabolism of exogenous lactate reveals it as a legitimate metabolic substrate in breast cancer. PLoS ONE.

[10] Sonveaux P (2008). Targeting lactate-fueled respiration selectively kills hypoxic tumor cells in mice. J. Clin. Invest..

[11] Pavlides S (2009). The reverse Warburg effect: Aerobic glycolysis in cancer associated fibroblasts and the tumor stroma. Cell Cycle.

[12] Faubert B (2017). Lactate metabolism in human lung tumors. Cell.

[13] Blatt S (2016). Lactate as a predictive marker for tumor recurrence in patients with head and neck squamous cell carcinoma (HNSCC) post radiation: A prospective study over 15 years. Clin. Oral. Investig..

[14] Brizel DM (2001). Elevated tumor lactate concentrations predict for an increased risk of metastases in head-and-neck cancer. Int. J. Radiat. Oncol. Biol. Phys..

[15] Poole RC, Halestrap AP (1993). Transport of lactate and other monocarboxylates across mammalian plasma membranes. Am. J. Physiol..

[16] Adijanto J, Philp NJ (2012). The SLC16A family of monocarboxylate transporters (MCTs)-physiology and function in cellular metabolism, pH homeostasis, and fluid transport. Curr. Top. Membr..

[17] Felmlee MA, Jones RS, Rodriguez-Cruz V, Follman KE, Morris ME (2020). Monocarboxylate transporters (SLC16): Function, regulation, and role in health and disease. Pharmacol. Rev..

[18] Jackson VN, Halestrap AP (1996). The kinetics, substrate, and inhibitor specificity of the monocarboxylate (lactate) transporter of rat liver cells determined using the fluorescent intracellular pH indicator, 2′,7′-bis(carboxyethyl)-5(6)-carboxyfluorescein. J. Biol. Chem..

[19] Morris ME, Felmlee MA (2008). Overview of the proton-coupled MCT (SLC16A) family of transporters: Characterization, function and role in the transport of the drug of abuse gamma-hydroxybutyric acid. Aaps J..

[20] Halestrap AP (2012). The monocarboxylate transporter family—Structure and functional characterization. IUBMB Life.

[21] Halestrap AP, Wilson MC (2012). The monocarboxylate transporter family—Role and regulation. IUBMB Life.

[22] Dhup S, Dadhich RK, Porporato PE, Sonveaux P (2012). Multiple biological activities of lactic acid in cancer: Influences on tumor growth, angiogenesis and metastasis. Curr. Pharm. Des..

[23] Payen VL, Mina E, Van Hee VF, Porporato PE, Sonveaux P (2020). Monocarboxylate transporters in cancer. Mol. Metab..

[24] Pinheiro C (2012). Role of monocarboxylate transporters in human cancers: State of the art. J. Bioenerg. Biomembr..

[25] Broer S (1998). Characterization of the monocarboxylate transporter 1 expressed in *Xenopus laevis* oocytes by changes in cytosolic pH. Biochem. J..

[26] Garcia CK, Goldstein JL, Pathak RK, Anderson RG, Brown MS (1994). Molecular characterization of a membrane transporter for lactate, pyruvate, and other monocarboxylates: Implications for the Cori cycle. Cell.

[27] Gill RK (2005). Expression and membrane localization of MCT isoforms along the length of the human intestine. Am. J. Physiol. Cell Physiol..

[28] Ritzhaupt A, Wood IS, Ellis A, Hosie KB, Shirazi-Beechey SP (1998). Identification and characterization of a monocarboxylate transporter (MCT1) in pig and human colon: its potential to transport l-lactate as well as butyrate. J. Physiol..

[29] Dimmer KS, Friedrich B, Lang F, Deitmer JW, Broer S (2000). The low-affinity monocarboxylate transporter MCT4 is adapted to the export of lactate in highly glycolytic cells. Biochem. J..

[30] Fox JEM, Meredith D, Halestrap AP (2000). Characterisation of human monocarboxylate transporter 4 substantiates its role in lactic acid efflux from skeletal muscle. J. Physiol..

[31] Ullah MS, Davies AJ, Halestrap AP (2006). The plasma membrane lactate transporter MCT4, but not MCT1, is up-regulated by hypoxia through a HIF-1alpha-dependent mechanism. J. Biol. Chem..

[32] Chen H (2010). Co-expression of CD147/EMMPRIN with monocarboxylate transporters and multiple drug resistance proteins is associated with epithelial ovarian cancer progression. Clin. Exp. Metastasis.

[33] Chen X (2019). Monocarboxylate transporter 1 is an independent prognostic factor in esophageal squamous cell carcinoma. Oncol. Rep..

[34] Pinheiro C (2010). Monocarboxylate transporter 1 is up-regulated in basal-like breast carcinoma. Histopathology.

[35] Pinheiro C (2008). Increased expression of monocarboxylate transporters 1, 2, and 4 in colorectal carcinomas. Virchows Arch..

[36] Pinheiro C (2009). The prognostic value of CD147/EMMPRIN is associated with monocarboxylate transporter 1 co-expression in gastric cancer. Eur. J. Cancer.

[37] Zhang G (2018). MCT1 regulates aggressive and metabolic phenotypes in bladder cancer. J. Cancer.

[38] Simoes-Sousa S (2016). Prognostic significance of monocarboxylate transporter expression in oral cavity tumors. Cell Cycle.

[39] Fleming JC (2019). HPV, tumour metabolism and novel target identification in head and neck squamous cell carcinoma. Br. J. Cancer.

[40] Curry JM (2013). Cancer metabolism, stemness and tumor recurrence: MCT1 and MCT4 are functional biomarkers of metabolic symbiosis in head and neck cancer. Cell Cycle.

[41] Sukeda A (2019). Expression of monocarboxylate transporter 1 is associated with better prognosis and reduced nodal metastasis in pancreatic ductal adenocarcinoma. Pancreas.

[42] Bola BM (2014). Inhibition of monocarboxylate transporter-1 (MCT1) by AZD3965 enhances radiosensitivity by reducing lactate transport. Mol. Cancer Ther..

[43] Doherty JR (2014). Blocking lactate export by inhibiting the Myc target MCT1 Disables glycolysis and glutathione synthesis. Cancer Res..

[44] Le Floch R (2011). CD147 subunit of lactate/H+ symporters MCT1 and hypoxia-inducible MCT4 is critical for energetics and growth of glycolytic tumors. Proc. Natl. Acad. Sci. USA.

[45] Zhu J (2014). Monocarboxylate transporter 4 facilitates cell proliferation and migration and is associated with poor prognosis in oral squamous cell carcinoma patients. PLoS ONE.

[46] Rave-Frank M (2016). Prognostic value of CXCL12 and CXCR4 in inoperable head and neck squamous cell carcinoma. Strahlenther. Onkol..

[47] Budczies J (2012). Cutoff Finder: A comprehensive and straightforward Web application enabling rapid biomarker cutoff optimization. PLoS ONE.

[48] Gross A, Ziepert M, Scholz M (2012). KMWin—A convenient tool for graphical presentation of results from Kaplan–Meier survival time analysis. PLoS ONE.

