# Supplementary material for: Monocarboxylate transporter-1 (MCT1) protein expression in head and neck cancer affects clinical outcome
Source: Sci Rep. 2021 Feb 25;11:4578. doi: 10.1038/s41598-021-84019-w (PMC7907348; doi:10.1038/s41598-021-84019-w)
Supplement: Supplementary file 1 — Supplementary Figures. [file 41598_2021_84019_MOESM1_ESM.docx]

**Monocarboxylate transporter-1 (MCT1) protein expression in head and neck cancer affects clinical outcome**

M Leu, J Kitz, Y Pilavakis, S Hakroush, HA Wolff, M Canis, S Rieken, MA Schirmer

**Supplemental Figure 1**. Immunohistochemical examples for MCT1 cell membrane staining. Observed staining intensities were 0 (absent, panel 1a), 1 (weak, panel 1b), and 2 (medium, panel 1c). Strong staining was not detected for MCT1.

| **1a**: No MCT1 staining |
| --- |
| 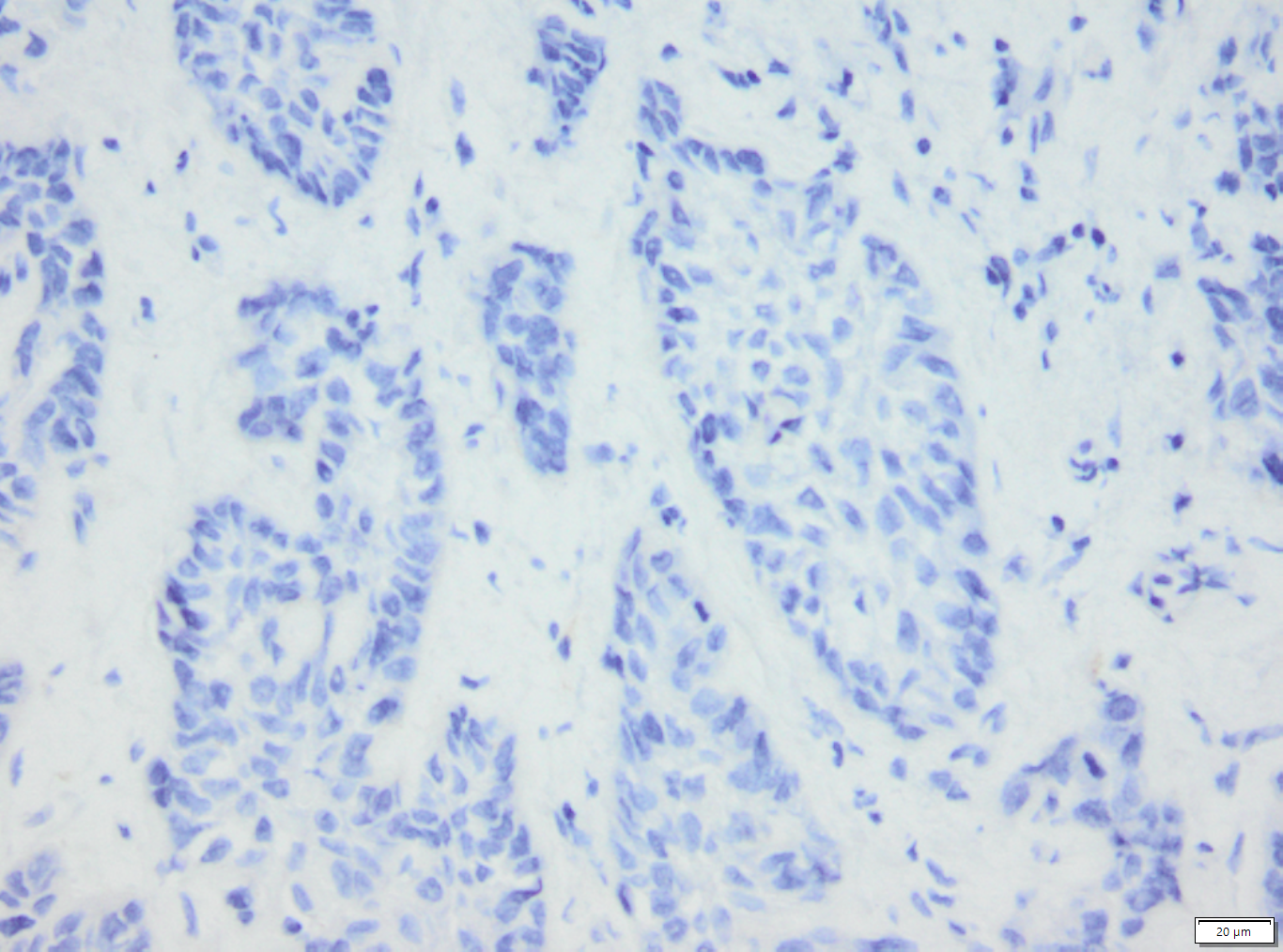 |
|  |
| **1b:** Weak MCT1 staining |
| 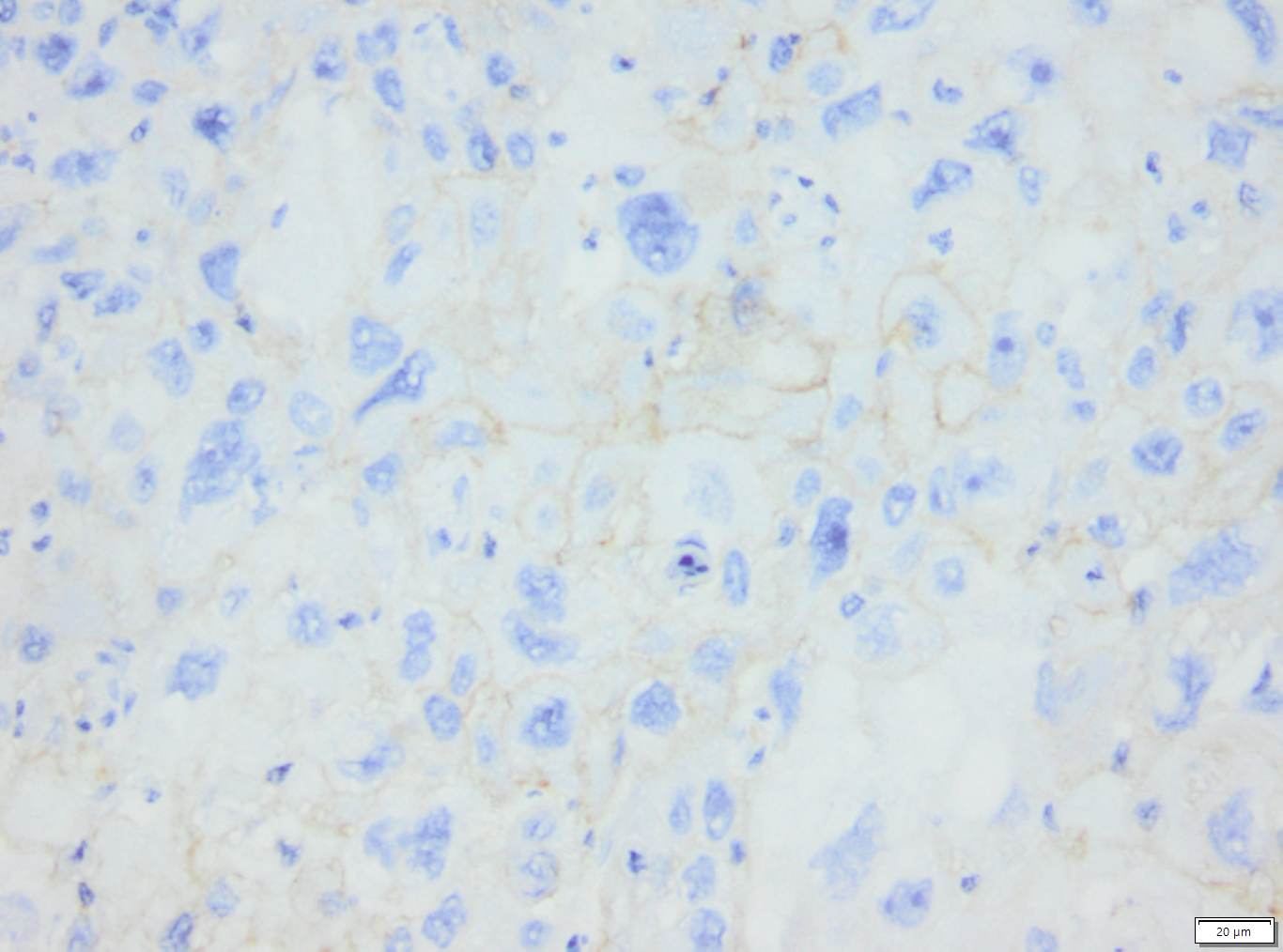 |
|  |
| **1c**: Medium MCT1 staining |
| 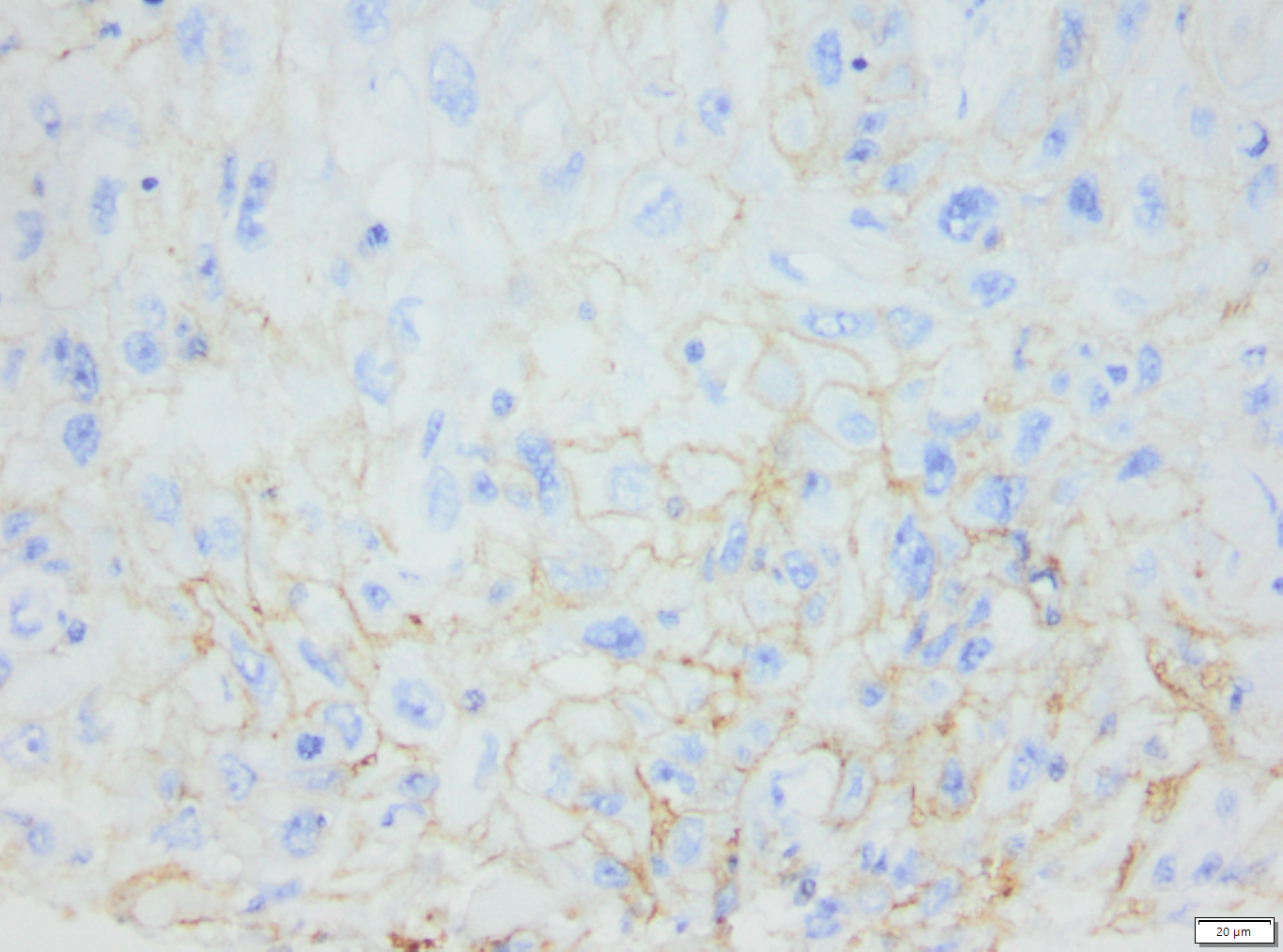 |

**Supplemental Figure 2**. Immunohistochemical examples for MCT4 cell membrane staining. Observed staining intensities were 0 (absent, panel 2a), 1 (weak, panel 2b), 2 (medium, panel 2c), and strong (panel 2d).

| **2a**: No MCT4 staining |
| --- |
| 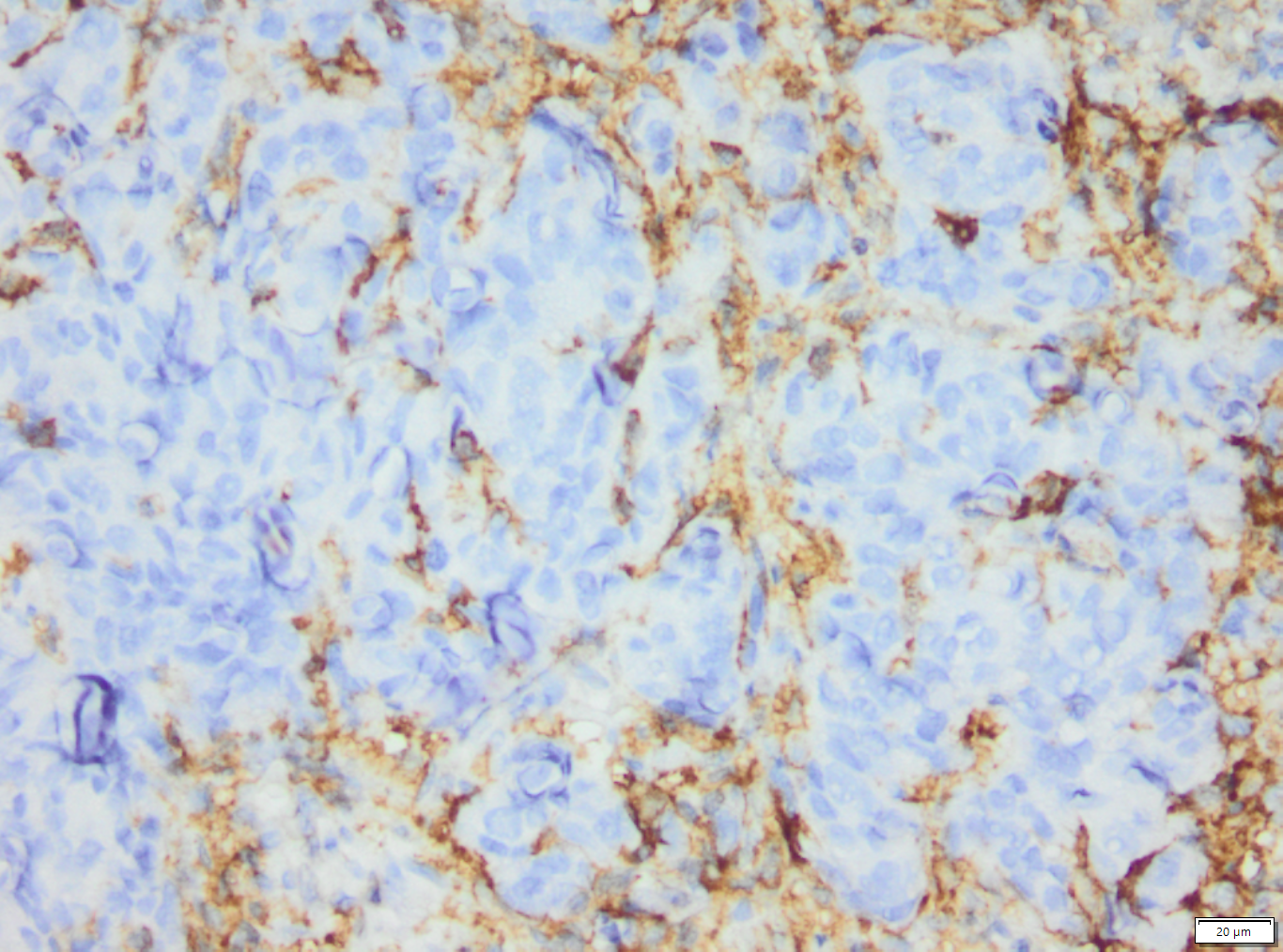 |
|  |
| **2b:** Weak MCT4 staining |
| 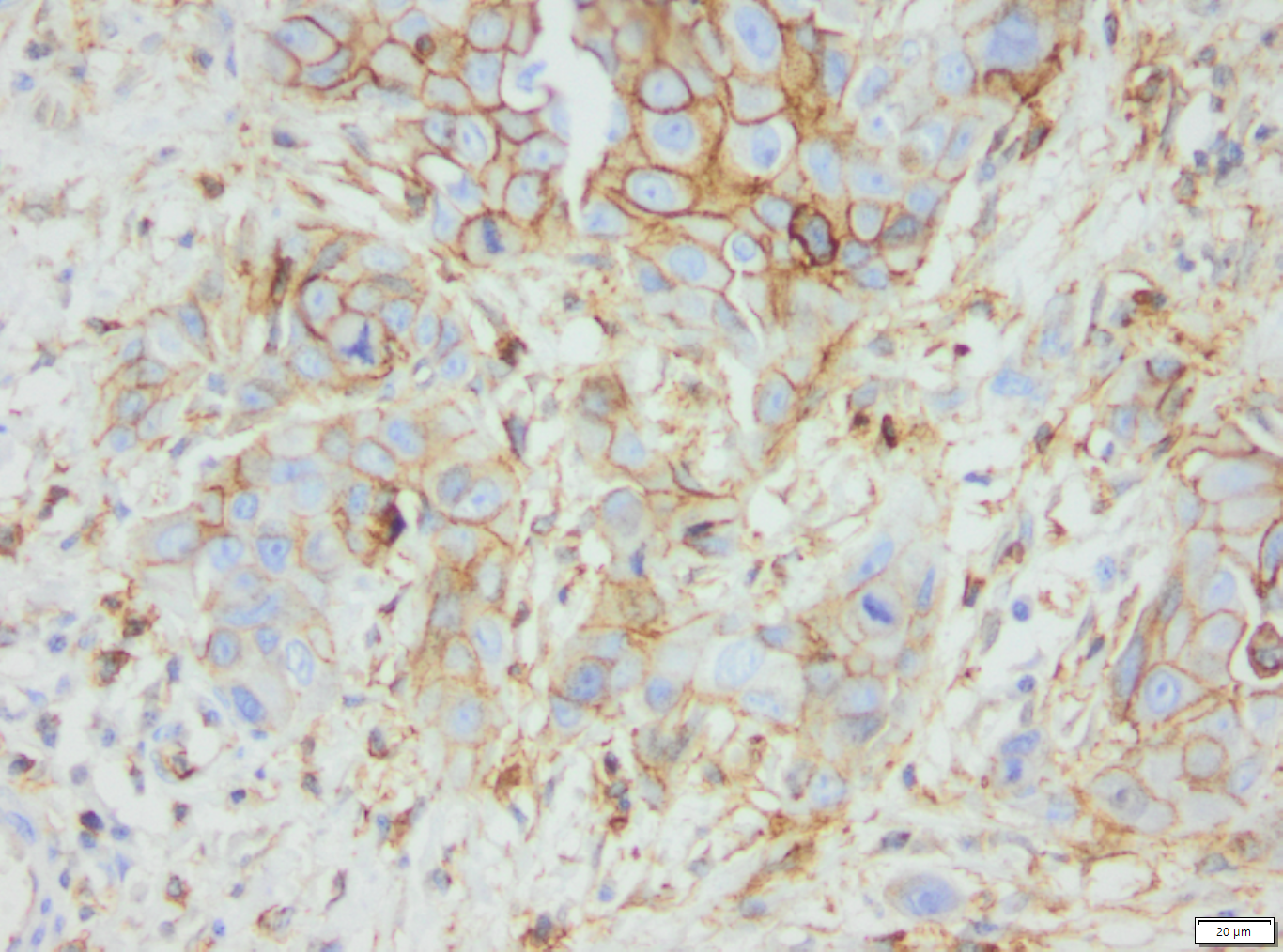 |
| **2c**: Medium MCT4 staining |
| 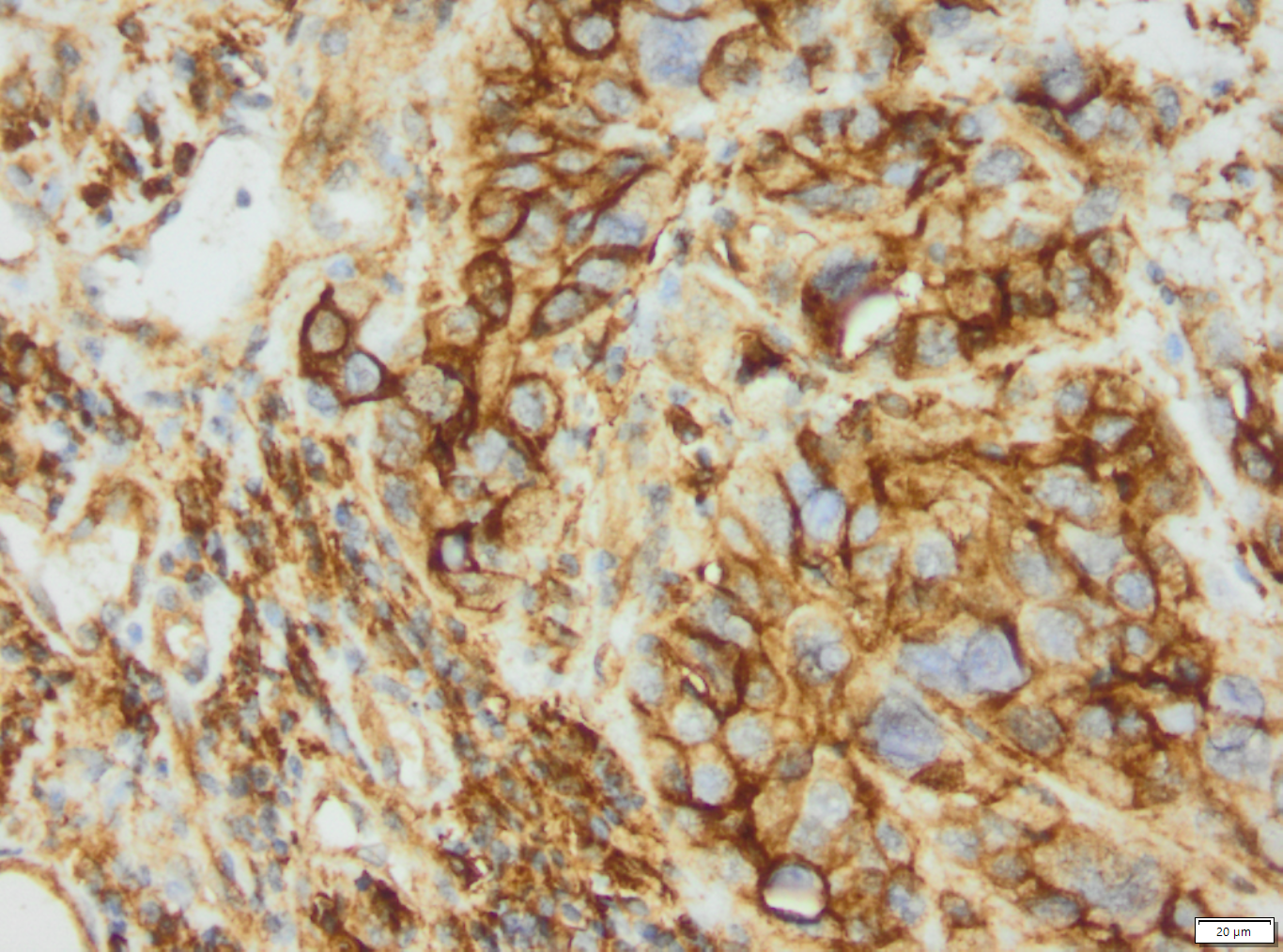 |
|  |
| **2d**: Strong MCT4 staining |
| 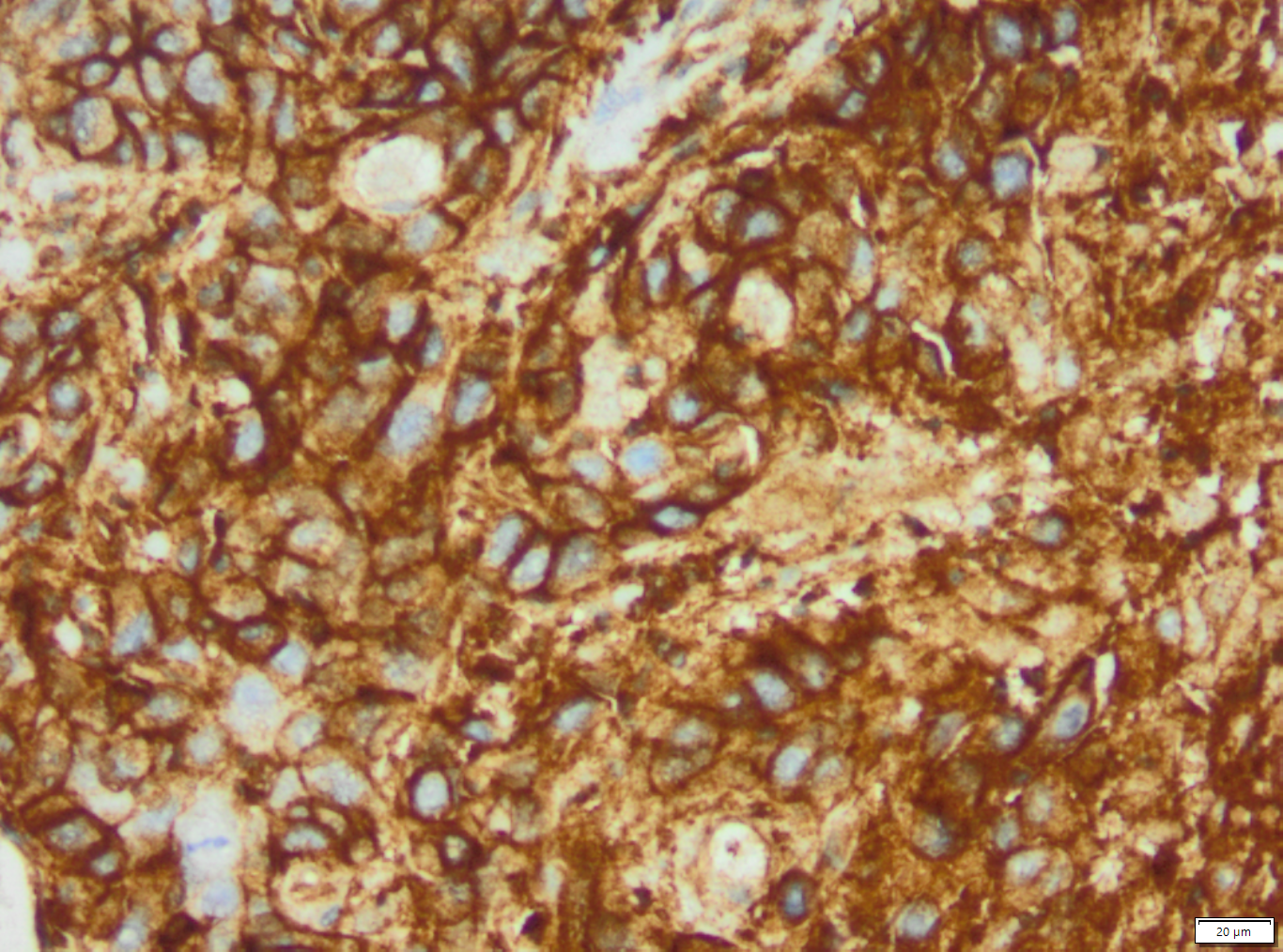 |
|  |
| **Supplemental Figure 3.** Distribution of membrane staining scores for MCT1(a) and MCT4(b) in the 82 sample cohort. For calculation of these scores please refer to Methods section.     |
|  |
|  |

**Supplemental Figure 4.** MCT effect on PFS and OS stratified by p16 status.

**Supplemental Figure 5.** Effect of p16 on PFS and OS stratified by MCT1 status.

**Supplemental Figure 6.** Subgroup of patients with p16+ and MCT1- with particular favorable outcome.
